# The complete chloroplast genome sequence of *Catalpa fargesii* (Bignoniaceae), a species endemic to China

**DOI:** 10.1080/23802359.2021.1914521

**Published:** 2021-06-21

**Authors:** Songzhi Xu, Wenjun Ma, Guijuan Yang

**Affiliations:** a School of Life Science, Nantong University, Nantong, China; b State Key Laboratory of Tree Genetics and Breeding; Key Laboratory of Tree Breeding and Cultivation of Forestry and Grassland Administration, Research Institute of Forestry, Chinese Academy of Forestry, Beijing, China

**Keywords:** Bignoniaceae, *Catapla fargesii*, phylogeny, chloroplast genome

## Abstract

*Catalpa fargesii* Bur. is endemic to China. Its complete chloroplast genome sequence was firstly reported in this study. The whole chloroplast genome of this species was 157765 bp in length including a pair of inverted repeat (IR, 30252 bp) regions separated by a small single copy (SSC, 12662 bp) and a large single copy (LSC, 84599 bp). The genome consisted of 134 genes, including 89 protein-coding genes, 8 rRNA and 37 tRNA genes. The phylogenetic analysis strongly supported that *C. fargesii* was closely related to *C. fargesii* f. *duclouxii* and *C. bungei*.

*Catalpa fargesii* Bur. is a deciduous tree, up to 25 m. It is endemic to China and mainly distributed in northwestern China. This species is an important economic tree species used as valuable timer for furniture, appliance and building because of its high density and hardness (Ma et al. 2021). *C. fargesii* also can be used horticulturally as garden and street trees for its large heart-shaped leaves and showy flowers (Ma et al. 2021). Besides, it can resist extreme drought and coldness. In the recent taxonomic revision of the genus *Catalpa*, *Catalpa fargesii* and *C. fargesii* var. *duclouxii* were treated as a single species, *C. catalpa* (Olsen and Kirkbride 2017). However, we recognized them to be different in several characters, including indumentum on young branches and leaves, leaf baldes, inflorescence, corolla, anthers. For instance, stellate or dendroid hairs of *C. fargesii* could differ it from *C. fargesii* var. *duclouxii* and *C. bungei*.

The sample of *C. fargesii* was collected from Maiji County, Gansu province, China (E 105.93920, N 34.44944). The voucher specimen (Ma WJ, HQ-1) was deposited at the Herbarium of Research Institute of Forestry, Chinese Academy of Forestry, Beijing, China (CAF) and DNA sample was properly stored at Key Laboratory of Tree Breeding and Cultivation of Forestry and Grassland Administration; Research Institute of Forestry, Chinese Academy of Forestry, Beijing, China. Total genomic DNA was extracted from the leaves dried with silica gel using the modified CTAB method of Doyle and Doyle (1987). For short-read sequencing, an ∼150 bp insert size pair-end library was constructed and sequenced using the Illumina HiSeq 2000 platform in the Sangon Biotech (Shanghai, China). A total of 5.5 Gb raw data were generated. By using SPAdes 3.13.0 (Bankevich et al. 2012) and Geneious 9.0.5 (http://www.geneious.com/), all contigs of the chloroplast genome sequence were spliced and assembled. Then, the Geneious 9.0.5 also was applied to annotate the complete chloroplast genome. The whole chloroplast genome of *Catalpa fargesii* f. *duclouxii* (MT783420) was used as a reference sequence. The complete chloroplast genome sequence has been deposited in GenBank with an accession number MW338733.

The circular chloroplast DNA of *C. fargesii* is 157,765 bp in length, and the average GC content was 37.43%. It consists of a typical quadripartite structure with two inverted repeats (IRs) of 30252 bp separated by a large single copy (LSC) of 84599 bp and a small single copy (SSC) of 12662 bp. The complete chloroplast genome of *C. fargesii* contains 134 unique genes, including 89 protein-coding genes, 37 tRNA genes and 8 rRNA genes. In these genes, 7 genes (2 tRNA genes and 5 protein-coding genes) contain one intron, whereas three genes (*ycf3*, *rpl2*, and *ndhB*) contain double introns.

To confirm the phylogenetic position of *C. fargesii* within the family of Bignoniaceae, additional 27 complete chloroplast genomes of Bignoniaceae were obtained from GenBank, and two species, *Schnabelia oligophylla* and *Callicarpa nudiflora* were used as the outgroups (Figure 1). The 29 complete chloroplast sequences were aligned by the MAFFT version 7 software (Katoh and Standley 2013). Phylogenetic analysis was conducted by using PAUP 4.0b10 (Swofford 2003) with 1000 bootstrap replicates and MrBayes 3.2.6 (Ronquist and Huelsenbeck 2003). The nucleotide substitution model was selected by the Akaike information criterion (AIC) in Modeltest 3.7 (Posada and Crandall 1998). For BI analysis, four chains of the Markov Chain Monte Carlo (MCMC) were run for 5,000,000 generations starting with a random tree, sampling one tree every 1000 generations. Majority-rule (>50%) consensus trees were generated after removing a 25% burn-in. Phylogenetic analysis results strongly supported that *C. fargesii* was closely related to a clade including *C. fargesii* f. *duclouxii* and *C. bungei* (MP-BS = 100, BI-PP = 1.00) (Figure 1). The phylogenetic relationship of Bignoniaceae recovered from this study is congruent with that of Olmstead et al. (2009).

**Figure 1.**
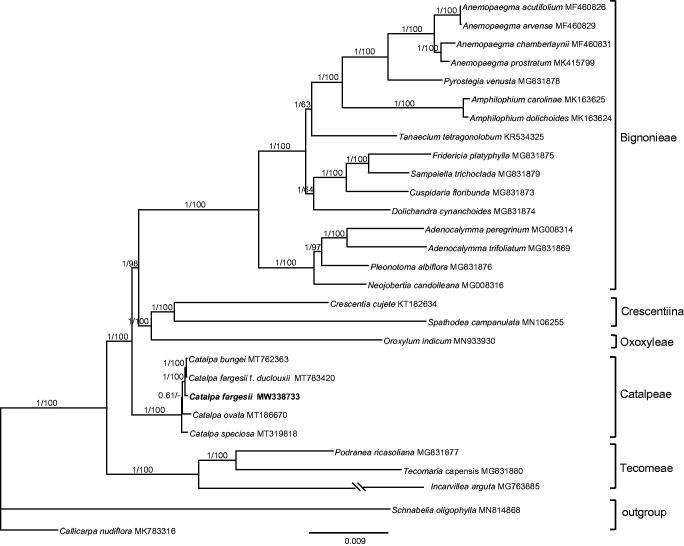
Maximum likelihood phylogram Bignoniaceae inferred from 29 chloroplast genomes. Numbers above branches indicated the maximum likelihood bootstrap support and the posterior probabilities, respectively.

## Data Availability

The data that support the finding of this study is available in GenBank at https://www.ncbi.nlm.nih.gov/ with an accession number MW338733. The associated BioProject, SRA, and Bio-Sample numbers are PRJNA715373, SAMN18347870, and SRR14027477, respectively.
